# Supplementary material for: Social and nutritional factors controlling the growth of honey bee (Apis mellifera) queens
Source: PLoS One. 2025 Feb 25;20(2):e0310608. doi: 10.1371/journal.pone.0310608 (PMC11856481; doi:10.1371/journal.pone.0310608)
Supplement: Attachment — Submitted filename: PONE-D-24-38555-ReviewerComments.pdf [file pone.0310608.s002.pdf]

PLOS ONE

The Social and Nutritional Factors Controlling the Growth of Honey Bee (Apis mellifera) Queens

--Manuscript Draft--

**Manuscript Number:**	PONE-D-24-38555
**Article Type:**	Research Article
**Full Title:**	The Social and Nutritional Factors Controlling the Growth of Honey Bee (Apis mellifera) Queens
**Short Title:**	Laboratory assay for honey bee queen rearing
**Corresponding Author:**	Hagai Yehoshua Shpigler, Dr.<br>ARO: Agricultural Research Organization<br>Rishon LeZion, ISRAEL
**Keywords:**	Honey bees; Honey bee queen; Grafting; Lab assay
**Abstract:**	The honey bee queen is essential for colony function, laying thousands of eggs daily and determining the colony's genetic composition. Beekeepers cultivate and trade queens to enhance colony health and productivity. Despite its significance, artificial queen rearing in foster queenless colonies has remained largely unchanged for over a century, offering limited control over the environmental conditions influencing larval development. In this study, we developed a laboratory-based method for queen bee rearing, establishing a protocol for rearing queens in cages in the lab under controlled environmental conditions. We first investigated the minimal number of worker bees required to rear a single queen and found that groups of 200 workers raise queens with comparable success and weight to those reared in colonies. As a proof of concept, we examined the impact of larval age on rearing success in our new system. We found that the age of the larvae affects the success rate of queen development and that younger larvae developed into heavier queens than older larvae, as published in the past using the traditional queen-rearing method. Additionally, we assessed the influence of pollen nutrition on queen-rearing success, finding that a high pollen concentration is crucial for queen development. These findings and the new method provide a foundation for studying queen bee development in a controlled environment and offer potential applications for improving queen-rearing practices.
**Order of Authors:**	Omer Kama
	Hagai Yehoshua Shpigler, Dr.
**Additional Information:**	
**Question**	**Response**
**Financial Disclosure**<br><br>Enter a financial disclosure statement that describes the sources of funding for the work included in this submission. Review the submission guidelines for detailed requirements. View published research articles from *PLOS ONE* for specific examples.<br><br>This statement is required for submission and **will appear in the published article** if the submission is accepted. Please make sure it is accurate.	The author(s) received no specific funding for this work.

**Funded studies**<br>Enter a statement with the following details:<br>• Initials of the authors who received each award<br>• Grant numbers awarded to each author<br>• The full name of each funder<br>• URL of each funder website<br>• Did the sponsors or funders play any role in the study design, data collection and analysis, decision to publish, or preparation of the manuscript?<br><br>Did you receive funding for this work?	
**Competing Interests**<br><br>Use the instructions below to enter a competing interest statement for this submission. On behalf of all authors, disclose any competing interests that could be perceived to bias this work—acknowledging all financial support and any other relevant financial or non-financial competing interests.<br><br>This statement is required for submission and **will appear in the published article** if the submission is accepted. Please make sure it is accurate and that any funding sources listed in your Funding Information later in the submission form are also declared in your Financial Disclosure statement.<br><br>View published research articles from *PLOS ONE* for specific examples.	The authors have declared that no competing interests exist.

Powered by Editorial Manager® and ProduXion Manager® from Aries Systems Corporation

**NO authors have competing interests**<br><br>Enter: *The authors have declared that no competing interests exist.*<br><br>**Authors with competing interests**<br><br>Enter competing interest details beginning with this statement:<br><br>*I have read the journal's policy and the authors of this manuscript have the following competing interests: [insert competing interests here]*<br><br>* typeset	
**Ethics Statement**<br><br>Enter an ethics statement for this submission. This statement is required if the study involved:<br><br>• Human participants<br>• Human specimens or tissue<br>• Vertebrate animals or cephalopods<br>• Vertebrate embryos or tissues<br>• Field research<br><br>Write "N/A" if the submission does not require an ethics statement.<br><br>General guidance is provided below. Consult the submission guidelines for detailed instructions. **Make sure that all information entered here is included in the Methods section of the manuscript.**	N/A

Powered by Editorial Manager® and ProduXion Manager® from Aries Systems Corporation

**Format for specific study types**<br><br>**Human Subject Research (involving human participants and/or tissue)**<br>• Give the name of the institutional review board or ethics committee that approved the study<br>• Include the approval number and/or a statement indicating approval of this research<br>• Indicate the form of consent obtained (written/oral) or the reason that consent was not obtained (e.g. the data were analyzed anonymously)<br><br>**Animal Research (involving vertebrate animals, embryos or tissues)**<br>• Provide the name of the Institutional Animal Care and Use Committee (IACUC) or other relevant ethics board that reviewed the study protocol, and indicate whether they approved this research or granted a formal waiver of ethical approval<br>• Include an approval number if one was obtained<br>• If the study involved *non-human primates*, add *additional details* about animal welfare and steps taken to ameliorate suffering<br>• If anesthesia, euthanasia, or any kind of animal sacrifice is part of the study, include briefly which substances and/or methods were applied<br><br>**Field Research**<br><br>Include the following details if this study involves the collection of plant, animal, or other materials from a natural setting:<br>• Field permit number<br>• Name of the institution or relevant body that granted permission	
**Data Availability**<br><br>Authors are required to make all data underlying the findings described fully available, without restriction, and from the time of publication. PLOS allows rare exceptions to address legal and ethical concerns. See the PLOS Data Policy and FAQ for detailed information.	Yes - all data are fully available without restriction

Powered by Editorial Manager® and ProduXion Manager® from Aries Systems Corporation

A Data Availability Statement describing where the data can be found is required at submission. Your answers to this question constitute the Data Availability Statement and **will be published in the article**, if accepted.<br><br>**Important:** Stating 'data available on request from the author' is not sufficient. If your data are only available upon request, select 'No' for the first question and explain your exceptional situation in the text box.<br><br>Do the authors confirm that all data underlying the findings described in their manuscript are fully available without restriction?	
**Describe where the data may be found in full sentences. If you are copying our sample text, replace any instances of XXX with the appropriate details.**<br><br>• If the data are **held or will be held in a public repository**, include URLs, accession numbers or DOIs. If this information will only be available after acceptance, indicate this by ticking the box below. For example: *All XXX files are available from the XXX database (accession number(s) XXX, XXX.).*<br>• If the data are all contained **within the manuscript and/or Supporting Information files**, enter the following: *All relevant data are within the manuscript and its Supporting Information files.*<br>• If neither of these applies but you are able to provide **details of access elsewhere**, with or without limitations, please do so. For example:<br><br>*Data cannot be shared publicly because of [XXX]. Data are available from the XXX Institutional Data Access / Ethics Committee (contact via XXX) for researchers who meet the criteria for access to confidential data.*<br><br>*The data underlying the results presented in the study are available from (include the name of the third party*	All relevant data are within the manuscript and its Supporting Information files.

and contact information or URL).<br>• This text is appropriate if the data are owned by a third party and authors do not have permission to share the data.<br><br>* typeset	
Additional data availability information:	Tick here if your circumstances are not covered by the questions above and you need the journal’s help to make your data available.

Powered by Editorial Manager® and ProduXion Manager® from Aries Systems Corporation

Manuscript

Click here to access/download;Manuscript;Kama and Shpigler 2023 Crowning a queen final.docx

~~The~~ Social and Nutritional Factors Controlling the Growth of Honey Bee (*Apis mellifera*) Queens

Omer Kama[1,2] and Hagai Y. Shpigler[1*]

[1] Department of Entomology, Agricultural Research Organization, The Volcani Institute, Rishon LeZion, Israel

[2] The Robert H. Smith Faculty of Agriculture, Food and Environment, The Hebrew University in Jerusalem, Rehovot, Israel

*****hagais@volcani.agri.gov.il

Abstract

This seems like an bit of an over estimation. Average is ~1200/day during peak honey and pollen flows.

The honey bee queen is essential for colony function, laying thousands of eggs daily and determining the colony's genetic composition. Beekeepers cultivate and trade queens to enhance colony health and productivity. Despite its significance, artificial queen rearing in foster queenless colonies has remained largely unchanged for over a century, offering limited control over the environmental conditions influencing larval development. In this study, we developed a laboratory-based method for queen bee rearing, establishing a protocol for rearing queens in cages in the lab under controlled environmental conditions. We first investigated the minimal number of worker bees required to rear a single queen and found that groups of 200 workers raise queens with comparable success and weight to those reared in colonies. As a proof of concept, we examined the impact of larval age on rearing success in our new system. We found that the age of the larvae affects the success rate of queen development and that younger larvae developed into heavier queens than older larvae, as published in the past using the traditional queen-rearing method. Additionally, we assessed the influence of pollen nutrition on queen-rearing success, finding that a high pollen concentration is crucial for queen development. These findings and the new method provide a foundation for studying queen bee development in a controlled environment and offer potential applications for improving queen-rearing practices.

Keywords: Honey bees, Honey bee queen, Grafting, Lab assay

Introduction

The honey bee (*Apis mellifera*) queen is the most important individual within the colony, as the reproductive success of the colony depends on her viability (1). Beekeepers typically replace their queens at least once every other year to maintain colony vigor (2). To meet this demand, new queens are raised annually. Artificial queen rearing was developed in the late 19th century in the U.S. by Gilbert Doolittle (3, 4), shortly after the establishment of the modern Langstroth method of beekeeping. This process is based on the principle that every diploid egg has the potential to develop into either a queen or a worker, depending on the social and physical environment of the developing larva (5). A new queen can be artificially reared by transferring young larvae from worker cells into large, vertically oriented queen cells in queenless colonies a process known as grafting (3). Queen breeders, ~~utilizing~~ specialized equipment and knowledge of honey bee biology. The queen-rearing industry includes thousands of breeders who grow millions of queens annually (6).

The honey bee queen has unique morphology, physiology, and behavior that support her ~~tasks~~ performance in the hive with maximal efficiency (1). Throughout her life cycle, the queen's behavior and physiology adapt to meet the demands of each stage. In the early stages of her life, after emerging from the pupa, the queen displays ~~high~~ aggressive behavior towards rival virgin queens, coinciding with the development of her venom sac and stinger. During her second week, the virgin queen embarks on nuptial flights, during which her flight muscles and navigation abilities fully develop (7, 8). Once the queen returns from her mating ~~flight~~, she remains in the hive, dedicating most of her time to egg-laying, venturing outside only during swarming events (9, 10). At this stage, her reproductive system and ovaries are fully developed, while her flight abilities and venom sac diminish. The success of a high-quality queen throughout all

life stages depends significantly on her genetic background and the environment in which she was raised (11, 12).

Honey bee colonies will rear new queens under three primary circumstances: in spring before swarming, as a replacement for a malfunctioning queen, or at emergence when the queen of the colony unexpectedly dies (1). The queen's development time is shorter than worker bees (10). Early emergence from the pupa is advantageous as it minimizes the period the colony is queenless, which is crucial for maintaining the colony's reproductive potential (13). Moreover, Competition among young queens is intense; the first queen to emerge typically attacks and kills rival queen pupae by stinging them before they can emerge (1). Queen breeders often raise multiple queens simultaneously within a single colony. To protect the pupae from the aggression of other queens, breeders separate the pupae around day ten of their development. A foster colony used for queen rearing typically contains tens of thousands of workers, frames with pupae, no young larvae, and no queen. The colony is well-nourished with honey and pollen to support the growth of queen larvae. Breeders usually introduce 40-60 larvae per cycle into the colony (1, 3). The queenless colony will then rear these larvae into queens as an emergency response. To ensure high-quality queens, breeders carefully construct the best possible foster colonies and select larvae from superior colonies. This method is highly efficient and meets market demands. However, breeders have no control over the nutrition that the colony gathers, and the foster colonies are exposed to environmental factors such as weather changes, predators, parasites, pesticides, and diseases including viruses (14). As a result, the rearing process sometimes fail.

The quality of a honey bee queen is influenced by both internal and external factors. Internally, it depends on the genetic background of the larvae and the genetic

source of the workers that rear the queen (12). Externally, environmental factors such as the age of the larvae, the type of nutrition provided, and the strength of the foster colony play critical roles (3, 12). Former studies shows that younger larvae, develop into larger and more fertile queens compared to older larvae (15). However, the control that a queen breeder has over the growth process is limited and generally ends after the larvae are introduced to the foster colony. In contrast, bumble bee breeding is conducted indoors, where conditions are tightly controlled to maximize success (16-18). This indoor breeding approach frees bumble bee breeders from the constraints of seasonality, enabling the year-round production of pollinators. However, this approach is not feasible for honey bees, since they live in large perennial colonies that cannot thrive indoors. This limitation reduces the ability of breeders to control environmental conditions, nutrition, and exposure to pathogens during the honey bee queen's development. Controlling the rearing conditions of honey bee queens can improve their quality and accelerate selective processes

In the current study, we introduce a new method for queen rearing under controlled environmental conditions. This method involves rearing queens using groups of bees housed in cages within a controlled environmental chamber, with a single larva introduced to each group for queen rearing. ~~The~~ Our method ~~allow the conduction of~~ permits large experiments ~~with~~ that are repeatable and reliable ~~replications~~. Our first experiment aimed to determine the minimum number of bees required to successfully rear a queen. We found that two hundred bees can reliably accomplish this task, but one hundred are not enough. We applied this method to investigate several research questions. As a proof of concept, we tested the effect of larval age on the development of queens within our system. As expected, we found that younger larvae developed into heavier queens as reported in the literature (19-21). Lastly, we explored the impact of nutrition on larval

do you mean the age of the larvae used to graft queens?

Queen breeders do control rearing environments for queens, are you referring to the environments (like seasonality) they cannot control? This is a very definitive statement with missing punctuation.

Perhaps highlight here that it's an indoor study i.e., rearing queens in the lab.

This is phrased exactly as in abstract.

These are not research questions is implied in previous sentence.

acceptance rates and the quality of the developing queens. By providing the bees with a pollen cake containing an elevated concentration of pollen, we demonstrated the significant effect of nutrition on queen growth. This new method and the findings presented here open the queen-rearing process to detailed investigation under controlled conditions, enabling further study of various factors affecting queen growth.

Materials and Methods

Bees

Bees from the apiary of the Volcani Center in Israel were used for the study. The experiments were conducted in the summer of 2023 and spring of 2024. Frames with emerging pupae were collected from the colonies and placed in a six-frame hive box in an acclimated room (34ºC ± 1, RH = 60% ± 5). The day old emerging bees were then collected into a plastic box and transferred to cages. The number of bees in each cage was estimated by weight using a scale with an accuracy of 0.01 g.

Cages

We used queen monitoring cages for this study (22). The cage size is 10 × 15 × 5 cm, with the back of a plastic frame. Each cage has four round holes designed to fit 5 ml tubes (diameter – 9 mm), two on the sides and two at the top. These holes are used for feeding and for the introduction of larval queen cups. The cages were supplied with pollen paste (90% pollen / 10% sugar water, 60%), a 5 ml tube of honey, and a 5 ml tube of water. The food was refreshed every other day. The cages were kept in an acclimated room with conditions resembling a normal hive (Temp: 34° ± 1°, RH: 60% ± 5%) for the entire experiment.

Queen rearing

colonies? Be consistent with referencing your stock colonies in your apiary.

Frames with young larvae were collected from the hives. Day-old larvae were collected from ~~the~~ cells using a grafting tool and gently transferred into queen-rearing cups (JZBZ queen bee cells) with a small droplet of royal jelly. The cups were introduced to ~~the~~ groups of bees in ~~the~~ cages through a hole at the top of the cage, one cell per cage. For control, cups from the same grafting session were introduced to a foster colony containing six frames of bees and no brood. The cells were monitored to estimate the success rate of larva acceptance ~~after 72 hours~~, queen cell ~~cup~~ capping, and the rate of ~~fully developed mature queens~~.

manufacturer? Manufacturer/source? queenless queen rearing; queen; throughout queen larval development; successful queen emergence.

Did you not experiment with the queen rearing process? Is this not why you completed experiment one? To see how many workers were needed to successfully raise a queen cell in the lab? Perhaps revise the narrative of your methods to more clearly communicate each experiment and the questions they address.

Queen weight

Since the queen's weight changes during her adult life, we weighed ~~the~~ queen s at the pupa stage when the environment does not affect her weight. The queen pupae were gently removed from the queen cells on day 10 after grafting, approximately one to two days before emergence. The pupae were weighed on an analytical scale to the nearest milligram to estimate the queen's weight. The pupae were then gently inserted back into the queen cups and sealed with wax. In experiment 2 (see below) the cups were left in the acclimated room for emergence, with each pupa in a separate cage. This manipulation did not affect the hatching success of the pupae. The emerging queens were collected and frozen at -20°C for further analysis.

Did you also do this for the queens that were raised in the control foster colony?

How did you test this?

What analysis? What experiment is this for?

Experiment 1: The Effect of the Number of Workers on Queen Development

The cages were populated with different numbers of workers. The number of workers in each cage was estimated by weighing the day-old bees added to the cage, with an estimation of ten bees per gram. The cages were populated with 5 g, 10 g, 20 g, or 30 g of bees representing 50, 100, 200 or 300 bees respectively. One day old larva, placed

What is your replication per treatment?

in a queen cup, was introduced to each cage and tracked for development. Three stages of development were monitored: larval acceptance after 3 days, the number of capped cells after one week, and queen emergence after 14 days. The acceptance and development of the larvae, as well as the weight of the queens, were measured as detailed above. The experiment was conducted using two different colonies.

Experiment 2: The Influence of Larval Age on Queen Development under Lab Conditions

Larvae of three different ages were used for queen rearing in cages. The cages were populated with 20 g of bees and fed *ad libitum* with honey and 90% pollen cake. To collect larvae of known age for grafting, a honey bee queen was caged in a queen excluder cage on a frame in a regular colony for 12 hours. The frame was checked for eggs at the end of the caging period, and the queen was released. The frame was kept in the cage for three days to ensure that the queen did not lay any more eggs on it. After three days, the frame was removed, and 12 larvae, aged 1-12 hours old, were used for queen rearing under controlled conditions. The frame was then returned to the queen excluder cage in the colony. A day later, on day four, the same frame was used for a second grafting of 24-36 hour-old larvae and returned to the colony. A final round of grafting was performed from the same frame for 48-60 hour-old larvae the next day. The acceptance and development of the larvae, as well as the weight of the queens, were measured as detailed above. The experiment was done with two different colonies.

Experiment 3: The Influence of Pollen Nutrition on Queen Development

The richness of the pollen cake was varied between the cages to test the effect of pollen nutrition on queen-rearing success. The bees were fed pollen paste at four different

all other treatments add to 100%, but this first one does not.

Protein and sugar?

levels of richness: Sugar water (60%) with no pollen (P0%), 30% pollen and 70% sugar water (P30%), 60% pollen and 40% sugar water (P60%), 90% pollen mixed with 10% sugar water (P90%). The cages were supplied with *ad libitum* honey ~~honey as a carbohydrate source~~. ~~One~~ day-old larvae were ~~larva was~~ grafted and introduced~~, one~~ to each cage. The acceptance and development of the larvae, as well as the weight of the developing queens, were measured as detailed above. The experiment was done with two different parent? colonies.

Data Analysis

What did you measure for queen development? Do you just mean the capping of the queen cell and queen emergence?

A ~~The~~ chi-square test of independence was used to compare ~~the success rate in~~ larval acceptance rate and queen development. A post hoc analysis was performed using a chi-square test of independence for each pair with FDR correction for multiple comparisons. Queen weight among treatments ~~The weight the queens~~ was compared using an ~~the~~ ANOVA ~~test~~. ~~The~~ data analyses ~~analysis~~ for this study was conducted using the Real Statistics Resource Pack software (Release 8.9.1). Copyright (2013 – 2023) Charles Zaiontz. www.real-statistics.com.

Can you clarify that you used this package and completed your statistics in Microsoft Excel.

If you collected survival data (i.e., yes and no, binomial data), why not do a glm binomial for each of the three time points (i.e., larval acceptance, cell capping, and queen emergence)?

Results

Experiment 1: The Effect of the Number of Workers on Queen Development.

We compared the queen-rearing success rate in groups of bees with different numbers of workers. The acceptance of the larvae was affected by the number of workers in the group. Fifty bees (50W) accepted only 25% of the larvae and did not grow any of the larvae to a mature queen (n = 20). Groups of 100 bees (100W) accepted 55% of the larvae on day three and grew 20% of them to maturity (n = 20). Two hundred bees (200W) accepted 69% of the larvae and grew 48% to queens (n = 19). Three hundred bees (300W) accepted 84% of the larvae and grew 63% to maturity. (Fig.1A, χ^2 test for independence, $\chi^2_{(6)} = 24.2$, $p < 0.001$). The success rate of queen-rearing in ~~a~~ control colony was 66% (n = 20), which was not different from the success rate of the 200W or 300W groups. ~~In a post hoc analysis, we found~~ the success rate of 50W ~~is~~ significantly lower than that of 200W and the 300W groups, and lower but not significant than the 100W (χ^2 test with FDR correction, $p < 0.05$). ~~The 100 group-reared queens were~~ not different from the ~~two and the three hundred groups~~ (χ^2 test with FDR correction, $p > 0.05$).

The weight of the queens was ~~also~~ affected by ~~the group size~~. ~~The~~ queens from the 100W group weighted 194 mg ± 1.0 (n = 2), queens of the 200W group ~~weight~~ 228 mg ± 3.8 (n = 8), and queens from the 300W group ~~weight~~ of 218 mg ± 2.6 (n = 10). The control colony raised queens with an average weight of 236 mg ± 2.6 (n = 13). The differences between the groups were significant (Fig 1B, One-way ANOVA, $F_{(3)} = 14.7$, $p < 0.001$). ~~In post hoc analysis we found a significant difference between the 100W group and all other groups (Tukey's post hoc test, p < 0.05). The colony's~~ queens

- (line 195, inserted above "a") the
- (line 197, inserted above "is") was
- (above line 200) Queens reared in the 100W cages were
- (below line 200) queens reared in the 200W and 300W cages
- (above line 202) upon adult emergence; the number of bees in the cages rearing the queens.
- (above line 203, 204) weighed; weighed; weighed
- (below line 208) , where queens raised by 100 bees weighed significantly less than all queens raised in all other treatments that produced viable queens.
- (margin) Perhaps report means and standard errors?
- (margin) So you had 79 cages (20 per treatment), plus 20 queen cells in a single foster colony? Did these all run at the same time? I.e., 100 larvae were grafted from the same two parent colonies? This all needs to be clarified in your methods.
- (margin) Define success rate, queen emergence or larval acceptance?
- (margin) Be consistent with your treatment references. Clearly define them above in your methods and use the same terminology or acronyms throughout.
- (margin) You include figures for both of these results. Can you refer to the figures and qualify the differences, instead of including all of the data from the figures in the text, deeming the figures unnecessary.

raised in the foster colony

were heavier than the 300W queens ($p < 0.05$) but not significantly different from the 200W queens. The 200W queens were similar in weight to the 300W queens.

Experiment 2: The Influence of the Larvae Age on Queen Development in Cages.

larval ages used to graft the queen larvae

queen

We tracked the acceptance and rearing success of larvae at three different ~~ages.~~ We used ~~groups of 200 bees~~ 200 bees per cage for this experiment. The accepted rate for young larvae at the age of 0-12 hours was 90%, ~~and~~ where 75% of accepted larvae developed into 75% developed to mature queens (n = 20); larvae at the age of 24-36 hours were accepted at a rate of 96% and 68% were fully developed (n = 25). Only 24% of the 48-60-hour-old larvae were accepted and 16% developed to queens (n = 25). The age of the larvae has a significant effect on the success rate of the development to queens (Fig 2A, Chi-square test for independence, $\chi^2_{(4)} = 37.3$, $p < 0.001$). In a post hoc analysis, we found the success rate of the 0-12h group and the 24-36h are higher than the 48-60h group (χ^2 test with FDR correction, $p < 0.05$). The 0-12h group is not different from the 24-36h in the success rate (χ^2 test with FDR correction, $p < 0.05$).

Perhaps report means and standard errors?

suggest revising throughout.

This is a vague statement. Suggest: Queen weight upon emergence was significantly impacted by the age of the larvae used to graft the queen cell (statistics).

The weight of the queens was affected by the age of the larva. Queens developed from 0-12h larvae weighed on average 234 mg ± 5 (n = 14) while 24-36h old weighed only 217 mg ± 6 (n = 15), the 48-60 h old larvae developed queens weighed only 206 mg ± 1 (n = 2). The difference between the groups is significant (Fig 2B, One-way ANOVA, $F_{(2)} = 4.8$, $p = 0.016$). In the Tukey post hoc test, the 0-12h larvae were heavier than the 24-36h larvae ($p = 0.03$). There ~~is~~ was no significant difference between the queens developed from the 24-36h and the 36-48h old larvae. Nonetheless, the sample size of the 36-48h group is low due to the low success rate of queen development in this group.

You include figures for both of these results. Can you refer to the figures and qualify the differences, instead of including all of the data from the figures in the text, deeming the figures unnecessary.

This can be written more concisely, especially with the figures.

Experiment 3: The influence of pollen nutrition on queen development

To test the effect of nutrition, we measured the success of queen-rearing by bees fed with pollen paste at different enrichment levels. ~~We used groups of 200 bees for this experiment.~~ We tested four levels of pollen paste enrichment (including pollen and sugar water): No pollen (P0%), P30%, P60% and P90% pollen paste. Removing the pollen from the cages (i.e., treatment P0%) resulted in zero acceptance of the larvae by the workers, and on day three, all the queen cups were empty and clean. Using 30% pollen paste, the bees accepted only a single larva out of 25 (4% success). At medium pollen paste of P60%, the bees accepted four out of 25 larvae (16%), and only two were reared to an adult queen. Using the heavy pollen paste of 90%, eighteen out 25 (72%) of the larvae were accepted by the workers on day three, and 16 were raised to mature queens. The difference between the groups is significant (Fig 3A, Chi-square test for independence, $\chi^2_{(6)} = 50.8$, $p < 0.001$). In a post hoc analysis, we found the success rate of the P90% group higher than all other groups (χ^2 test with FDR correction, $p < 0.05$). The P0%, P30% and P60% are not different from each other in the success rate of queen rearing (χ^2 test with FDR correction, $p > 0.05$).

move to methods

Same comments as previous. Use your accompanying figures. State the results, qualify them, and refer to figure.

This can be written more concisely.

Suggestions on previous results paragraphs apply here too!

The average weight of the queen in the P90% group was 237mg ± 4 (n = 13). The average weight of the queens in the P60% group was 228mg ± 2 (n = 2), and the single queen developed at the P30% group weight was 233mg (Fig 3B). Due to the low rearing rate of the queens in the low pollen groups, the differences between the groups were not compared.

I don't think a figure is warranted here.

Suggestions on previous results paragraphs apply here too!

Discussion

The process of queen development depends on various biotic and abiotic factors (23-25). Queen rearing is a social effort, and the workers' decision to rear a queen is made collectively. In this study, we developed a new protocol to investigate the environmental and social conditions required for successful queen development. Our findings demonstrate that the workers' decision to rear a queen is influenced by several social and physiological factors, including the number of bees in the group, their nutritional state, and the age of the larvae.

We found that a minimum number of worker bees is necessary to successfully rear a queen from a day-old larva. Fifty workers accepted only a few larvae and nurtured them until day three, but none successfully reared a queen. One hundred bees accepted about half of the larvae, but only a few were raised to maturity. Groups of 200 or 300 workers succeeded in rearing queens at rates not significantly different, compared to a full colony in the current study and consistent with data from other studies (3, 26-28). We assessed queen quality using wet weight of the queens, a common metric for predicting queens' reproductive potential and hive performance (11, 29-33). The weight of queens reared in the lab by 200 bees was comparable to that of queens reared in traditional colony-based systems. This finding suggests that the indoor queen rearing method developed in this study is comparable in quality to colony-based rearing and that 200 bees is the minimal reliable number required for successful queen rearing.

You're re-reporting your results here.

What is this other data? Others have tested the minimum number of bees required to raise a queen before? These cited papers, do not appear to test this result.

This is more of a method statement.

Define quality. You did not test queen mating or introduction into colonies, so this is a pretty big leap given the very different rearing environments.

The collective decision of the bees to participate in queen rearing is dynamic, with bees adjusting their behavior to changing conditions. For example, eight bees can successfully rear a queen from a four-day-old larva (34), and even a single worker can care for a four-day-old larva (35). However, fifty bees are insufficient to rear a queen

from a one-day-old larva. Interestingly, in half of the cases, the bees began the rearing process and accepted the larva, but later abandoned it. This suggests that the decision to rear a queen depends on a complex interaction between the number of workers and the age of the larva. How the workers assess their number in the cage is a puzzling question that should be further studied. Based on the findings of our first experiment, we continued to investigate the parameters affecting queen rearing in groups of two hundred bees, a practical and easily replicable method. As our protocol became more refined, our success rate in queen-rearing increased in the next experiments.

It would have been interesting to compare the same number of bees in colony caring for a queen cell through some kind of exclusion or cage manipulation.

In our second experiment, we tested the effect of larval age on the quality of the developed queen using two hundred bees in each cage. Several studies have shown that the age of the larvae at grafting affects the quality of the developing queens. Younger larvae tend to develop into higher-quality queens in traditional rearing in foster colonies (15, 19, 33, 36, 37) and in in vitro hand-rearing (25). Our findings support these studies, as we found that larvae aged 0-12 hours developed into heavier queens compared to older larvae. We also demonstrated that the acceptance rate of larvae depends on their age, with larvae older than 48 hours rarely developing into queens (19). The findings of this experiment provide a proof of concept for the new laboratory method of queen rearing, as we were able to consistently replicate former results.

citation?

define quality

italics

what former results?

The nutrition available to the workers also influences their decision to accept larvae for queen rearing. When we removed all pollen from the cages, the bees did not accept any of the larvae. A low-protein diet resulted in a low success rate in queen rearing, with only well-nourished workers accepting the task of queen rearing. This finding supports the hypothesis that the nutritional state of the bees is a crucial factor in their decision to rear a queen . The nutritional condition of the workers affects many functions of the

space before period

Discuss in the context of the literature? The nutritional needs of bees to rear any larvae is well documented.

new topic, new paragraph?

bees, including the queen's egg-laying capacity (22), learning and memory (38) foraging behavior (39) as well as their physiology and immunity (40). Our new method can also be used to test the effects of other nutritional factors on the success of queen rearing, such as different types of pollen, pollen supplements, and pesticide residuals (41-43).

Queen rearing is a major tool for bee stock selection and improvement. The lack of control over queen-rearing conditions has turned this process into a "black box," where the breeder's influence on the queen's development has been limited. Rearing queens under artificially controlled environmental conditions can help extend the queen-rearing season (44). Additionally, isolating developing queens from the hive environment can reduce the impact of pathogens, such as the black queen cell virus, on the success of queen rearing (45). This method can also be employed to test the effects of new pesticides on honey bee health (46, 47). We anticipate that further studies using this new method will shed light on queen development and worker-rearing behavior.

I don't completely buy some of these arguments. Simply because the success of rearing queens in the lab with your method, relies 100% on having access to bee larvae in the field. Extending the queen rearing season via lab rearing does not seem viable under the dependent scenario on access to larvae in the field.

Acknowledgments:

You declared no funding!

We want to thank the Volcani Institution for providing ~~the~~ facilities and funding for this study. We thank Dr. Victoria Soroker for help with the experiment design and the beekeeper Assaf Otmy for help with the bees.

Figures legend:

This figure as written does not report success rate. It reports the number of larve that were accepted, rejected, and capped.

Fig 1: The effect of group size on queen development. A. ~~The success rate of queen growth by groups of bees of different sizes.~~ The column represents the number of larvae rejected by the bees (White) accepted as larvae but were not ~~caped~~ capped and pupated (Grey) capped queen pupa (Black). ~~The *p-value* is a result of a χ^2 test of independence,~~ Columns with different letters are significantly different ~~in post hoc test~~ following a chi square (χ^2 test with FDR correction $p < 0.05$). **B.** The weight of queen pupae of grown by nurse bee groups of bees of different sizes or by a full colony. The column represents the ~~average~~ mean ± SE~~; the *p-value* is a result of the One-way ANOVA test.~~ Columns with different letters are significantly different in the Tukey pos-hoc test (report ANOVA stats $p < 0.05$).

I think a revised figure would work better here, where success rate was your response variable, and the three developmental time points (i.e., larval acceptance, cell capping, and queen emergence) were reported as barplots side-by-side for each treatment.

I think you should revise all of your "success rate" figures as appropriate to suggestion above.

Fig 2: The effect of larval age on queen development ~~under controlled environment~~. The success rate (A) and the weight (B) of ~~queen~~ queens reared by 200 bees from larvae from the same source at three different ages (at time of grafting?): 0-12 hours, 24-36 hours, 48-60 hours. Detailed as at Fig 1. (Yeah, this isn't recommended.)

Fig 3: The effect of pollen nutrition on queen development. The success rate (A) and the weight (B) of queen growth by groups of 200 bees fed with different pollen paste concentration. Detailed as at Fig 1. (This tells the reader nothing. What details!?!?!)

References

1. Laidlaw HH, Page RE. Queen rearing and bee breeding. Cheshire, CT, USA: Wicwas Press; 1997.
2. Bienkowska M, Los A, Wegrzynowicz P. Honey bee queen replacement: An analysis of changes in the preferences of Polish beekeepers through decades. Insects. 2020;11(8):1-13.
3. Buechler R, Andonov S, Bienefeld K, Costa C, Hatjina F, Kezic N, et al. Standard methods for rearing and selection of *Apis mellifera* queens. J Apic Res. 2013;52(1):1-16.
4. Doolittle G. Scientific queen-rearing as practically applied: Being a method by which the best of queen-bees are reared in perfect accord with nature's ways. Am Bee J. 1915;55:126.
5. Lyko F, Foret S, Kucharski R, Wolf S, Falckenhayn C, Maleszka R. The honey bee epigenomes: Differential methylation of brain DNA in queens and workers. PLoS Biol. 2010;8(11):e1000506.
6. Cobey SW, Sheppard WS, Tarpy DR. Status of breeding practices and genetic diversity in domestic US honey bees. In: Sammataro D, Yoder JA, editors. Honey bee colony health: Challenges and sustainable solutions. Boca Raton, FL, USA: CRC Press; 2012. p. 25-36.
7. Uzunov A, Andonov S, Dahle B, Kovacic M, Presern J, Aleksovski G, et al. Standard methods for direct observation of honey bee (*Apis mellifera* L.) nuptial flights. J Apic Res. 2024;63(1):65-75.
8. Simone-Finstrom M, Tarpy DR. Honey bee queens do not count mates to assess their mating success. J Insect Behav. 2018;31(2):200-9.
9. Shpigler HY, Yaniv A, Gernat T, Robinson GE, Bloch G. The influences of illumination regime on egg-laying rhythms of honey bee queens. J Biol Rhythms. 2022;37(6):609-19.
10. Winston ML. The biology of the honey bee. Cambridge, MA, USA: Harvard University Press; 1987.
11. Akyol E, Yeninar H, Kaftanoglu O. Live weight of queen honey bees (*Apis mellifera* L.) predicts reproductive characteristics. J Kans Entomol Soc. 2008;81(2):92-100.
12. Hatjina F, Bienkowska M, Charistos L, Chlebo R, Costa C, Drazic MM, et al. A review of methods used in some European countries for assessing the quality of honey bee queens through their physical characters and the performance of their colonies. J Apic Res. 2014;53(3):337-63.
13. Winston ML, Marceau J, Higo H, Cobey S. Honey bee pheromones do not improve requeening success. Am Bee J. 1998;138(12):900-3.
14. Amiri E, Strand MK, Tarpy DR, Rueppell O. Honey bee queens and virus infections. Viruses. 2020;12(3):1-23.
15. Rangel J, Keller JJ, Tarpy DR. The effects of honey bee (*Apis mellifera* L.) queen reproductive potential on colony growth. Insectes Soc. 2013;60(1):65-73.
16. Hughes MJ. Commercial rearing of bumble bees. In: Matheson A, editor. Bumble bee production, pollination, and conservation. Cardiff, UK: International Bee Research Association; 1996. p. 40-7.
17. Goulson D. Bumblebees: Their behaviour and ecology. New York, NY, USA: Oxford University Press; 2003.

18. Giacomini JJ, Connon SJ, Marulanda D, Adler LS, Irwin RE. The costs and benefits of sunflower pollen diet on bumble bee colony disease and health. Ecosphere. 2021;12(7):e03626.
19. Rehman N, Anjum S, Qureshi N, Khan M, Albasher G, Kaleem M, et al. The effect of larval age, and wet and dry grafting, on the rearing of queen bees using the Doolittle grafting method. Entomol Res. 2024;54:1-10.
20. Mahbobi A, Farshineh-Adl M, Woyke J, Abbasi S. Effects of the age of grafted larvae and the effects of supplemental feeding on some morphological characteristics of Iranian queen honey bees (*Apis mellifera* meda Skorikov, 1929). J Apic Sci. 2012;56(2):93-8.
21. Woyke J. Correlations between the age at which honeybee brood was grafted, characteristics of the resultant queens, and results of insemination. J Apic Res. 1971;10(1):45-55.
22. Fine JD, Shpigler HY, Ray AM, Beach NJ, Sankey AL, Cash-Ahmed A, et al. Quantifying the effects of pollen nutrition on honey bee queen egg laying with a new laboratory system. PLoS One. 2018;13(9):e0203444.
23. Tarpy DR, Talley E, Metz BN. Influence of brood pheromone on honey bee colony establishment and queen replacement. J Apic Res. 2020;60(2):220-8.
24. Johnson RM, Percel EG. Effect of a fungicide and spray adjuvant on queen-rearing success in honey bees (Hymenoptera: Apidae). J Econ Entomol. 2013;106(5):1952-7.
25. De Souza DA, Hartfelder KH, Tarpy DR. Effects of larval age at grafting and juvenile hormone on morphometry and reproductive quality parameters of in vitro reared honey bees (Hymenoptera: Apidae). J Econ Entomol. 2019;112(5):2030-9.
26. da Silva E, da Silva R, Chaud-Netto J, Moreti A, Otsuk I. Influence of management and environmental factors on mating success of Africanized queen honey bees. J Apic Res. 1995;34:169-75.
27. Contreras-Martinez C, Contreras-Escareño F, Macias-Macias J, Tapia-Gonzalez J, Petukhova T, Guzman-Novoa E. Effect of different substrates on the acceptance of grafted larvae in commercial honey bee (*Apis mellifera*) queen rearing. J Apic Sci. 2017;61(2):245-51.
28. Genc F, Emsen B, Dodologlu A. Effects of rearing period and grafting method on the queen bee rearing. J Appl Anim Res. 2005;27(1):45-8.
29. Medina LM, Goncalves LS. Effect of weight at emergence of Africanized (*Apis mellifera* L.) virgin queens on their acceptance and beginning of oviposition. Am Bee J. 2001;141(3):213-5.
30. Alqarni AS, Balhareth HM, Owayss AA. Queen morphometric and reproductive characters of Apis mellifera jemenitica, a native honey bee to Saudi Arabia. Bull Insectol. 2013;66(2):239-44.
31. De Souza DA, Wang Y, Kaftanoglu O, De Jong D, Amdam GV, Goncalves LS, et al. Morphometric identification of queens, workers and intermediates in in vitro reared honey bees (*Apis mellifera*). PLoS One. 2015;10(4):e0123663.
32. Abd Al-Fattah MA, El-Din HAS, Ibrahim YY. Factors affecting the quality of mated honey bee queens stored for different periods in queen-right bank colonies: Effect of cage level and position on holding frame. J Apic Res. 2016;55(4):284-91.
33. Kahya Y, Gencer HV, Woyke J. Weight at emergence of honey bee (*Apis mellifera* caucasica) queens and its effect on live weights at the pre and post mating periods. J Apic Res. 2008;47(2):118-25.

34. Shpigler HY, Robinson GE. Laboratory assay of brood care for quantitative analyses of individual differences in honey bee (*Apis mellifera*) affiliative behavior. PLoS One. 2015;10(11):e0143183.
35. Shpigler HY, Saul MC, Corona F, Block L, Ahmed AC, Zhao SD, et al. Deep evolutionary conservation of autism-related genes. Proc Natl Acad Sci U S A. 2019;116(35):17600-5.
36. Njeru L, Raina S, Kutima H, Salifu D, Cham D, Kimani J, et al. Effect of larval age and supplemental feeding on morphometrics and oviposition in honey bee *Apis mellifera* scut queens. J Apic Res. 2017; 56(3): 183-89.
37. Dedej S, Hartfelder K, Aumeier P, Rosenkranz P, Engels W. Caste determination is a sequential process: Effect of larval age at grafting on ovariole number, hind leg size and cephalic volatiles in the honey bee (*Apis mellifera* carnica). J Apic Res. 1998;37(3):183-90.
38. Arien Y, Dag A, Zarchin S, Masci T, Shafir S. Omega-3 deficiency impairs honey bee learning. Proc Natl Acad Sci U S A. 2015;112(51):15761-6.
39. Pernal SF, Currie RW. The influence of pollen quality on foraging behavior in honeybees (*Apis mellifera* L.). Behav Ecol Sociobiol. 2001;51(1):53-68.
40. Corona M, Branchiccela B, Alburaki M, Palmer-Young E, Madella S, Chen Y, et al. Decoupling the effects of nutrition, age, and behavioral caste on honey bee physiology, immunity, and colony health. Front Physiol. 2023;14:1-16.
41. Hendriksma HP, Shafir S. Honey bee foragers balance colony nutritional deficiencies. Behav Ecol Sociobiol. 2016;70(4):509-17.
42. Retschnig G, Rich J, Crailsheim K, Pfister J, Perreten V, Neumann P. You are what you eat: Relative importance of diet, gut microbiota and nestmates for honey bee, *Apis mellifera*, worker health. Apidologie. 2021;52(3):632-46.
43. Milone JP, Tarpy DR. Effects of developmental exposure to pesticides in wax and pollen on honey bee (*Apis mellifera*) queen reproductive phenotypes. Sci Rep. 2021;11(1):1-11.
44. Taber S III, Poole HK. Rearing and mating of queen and drone honey bees in winter. Am Bee J. 1974;114(1):18-9.
45. Kevill JL, Lee K, Goblirsch M, McDermott E, Tarpy DR, Spivak M, et al. The pathogen profile of a honey bee queen does not reflect that of her workers. Insects. 2020;11(6):1-11.
46. Zhu W, Schmehl D, Mullin C, Frazier J. Four common pesticides, their mixtures and a formulation solvent in the hive environment have high oral toxicity to honey bee larvae. PLoS One. 2014;9(6):e100162.
47. Gregorc A, Ellis J. Cell death localization in situ in laboratory reared honey bee (*Apis mellifera* L.) larvae treated with pesticides. Pestic Biochem Physiol. 2011;99(2):200-7

Figure 1

Click here to access/download;Figure;Kama and Shpigler Fig1.pdf

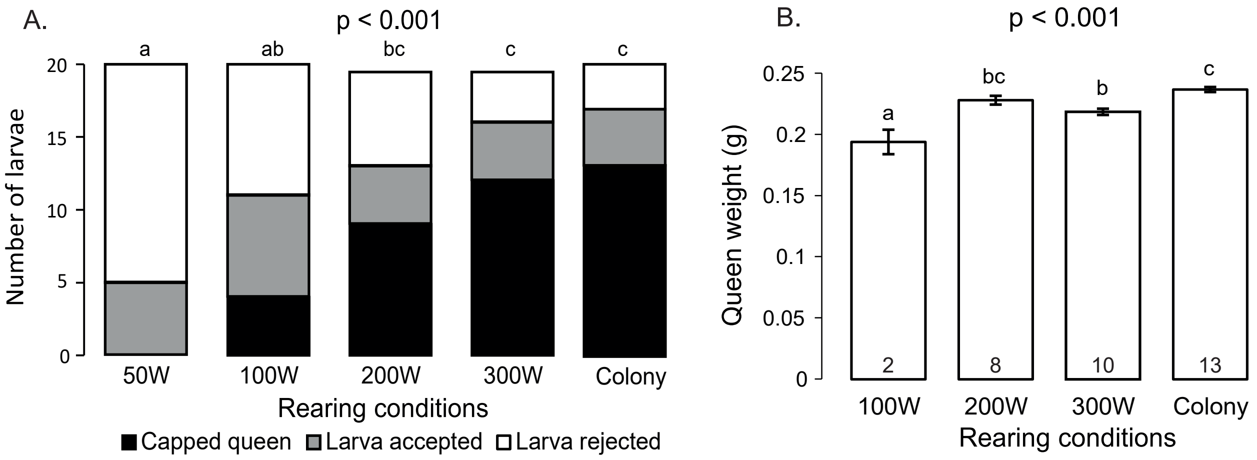

rearing conditions = treatment?

what about queen emergence? did all queens emerge from queen cell after capping?

Figure 2

Click here to access/download;Figure;Kama and Shpigler Fig2.pdf

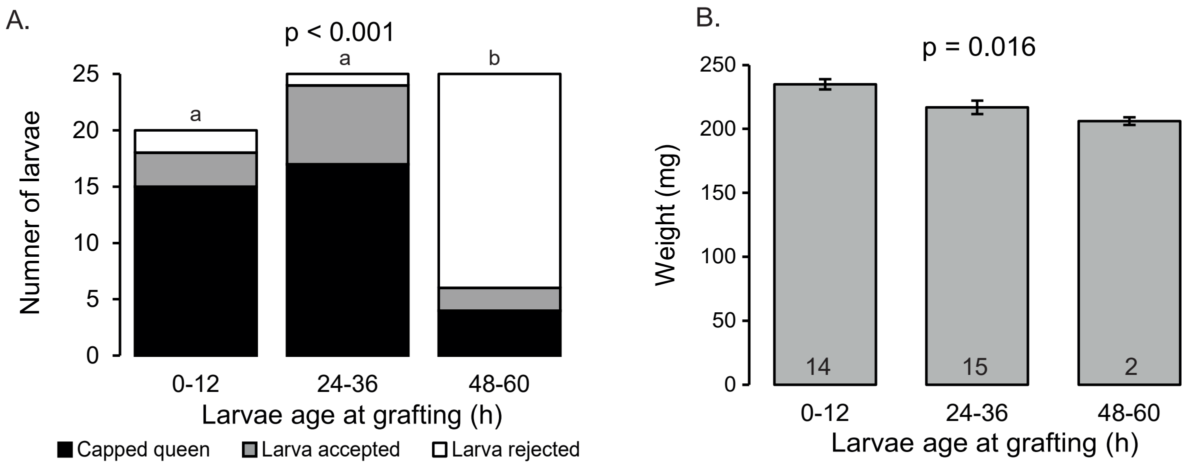

Larval Age at
Grafting (hours)

Queen emergence?

Figure 3

Click here to access/download;Figure;Kama and Shpigler Fig3.pdf

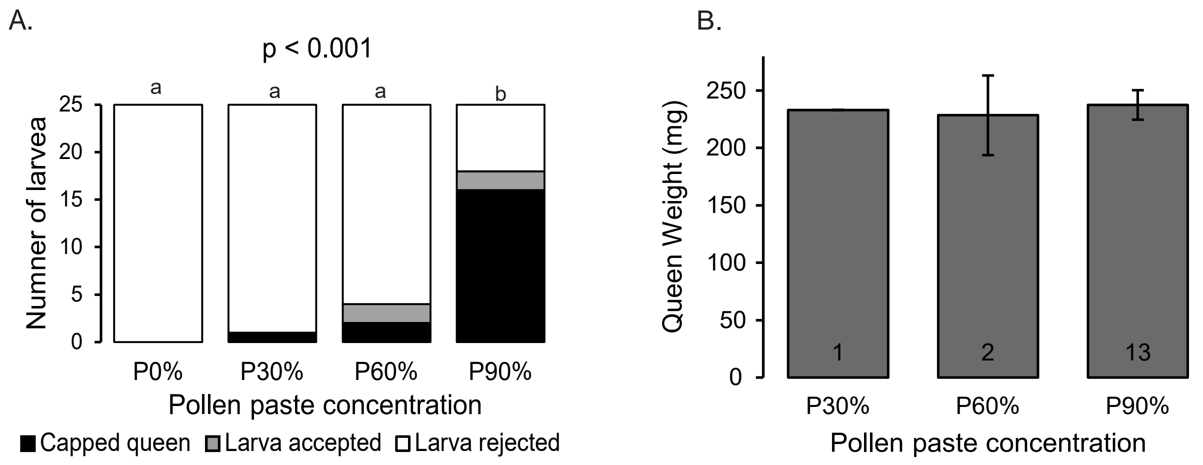

Queen emergence?

x label: percent pollen ?

Click here to access/download
Supporting Information
Kama and Shpigler Supplementary material.xlsx
